# Structural variability and niche differentiation in the rhizosphere and endosphere bacterial microbiome of field-grown poplar trees

**DOI:** 10.1186/s40168-017-0241-2

**Published:** 2017-02-23

**Authors:** Bram Beckers, Michiel Op De Beeck, Nele Weyens, Wout Boerjan, Jaco Vangronsveld

**Affiliations:** 1 Centre for Environmental Sciences, Hasselt University, Agoralaan building D, B-3590 Diepenbeek, Belgium; 2 Current address: Department of Biology, Lund University, Ecology Building, SE-22 362 Lund, Sweden; 3 Department of Plant Systems Biology, VIB, Technologiepark 927, B-9052 Ghent, Belgium; 4 Department of Plant Biotechnology and Bioinformatics, UGent, Technologiepark 927, B-9052 Ghent, Belgium

**Keywords:** *Populus tremula × Populus alba*, Bacterial microbiome, Rhizosphere, Endosphere, Microbiome niche differentiation, 16S rRNA amplicon pyrosequencing

## Abstract

**Background:**

The plant microbiome represents one of the key determinants of plant health and productivity by providing a plethora of functional capacities such as access to low-abundance nutrients, suppression of phytopathogens, and resistance to biotic and/or abiotic stressors. However, a robust understanding of the structural composition of the bacterial microbiome present in different plant microenvironments and especially the relationship between below-ground and above-ground communities has remained elusive. In this work, we addressed hypotheses regarding microbiome niche differentiation and structural stability of the bacterial communities within different ecological plant niches.

**Methods:**

We sampled the rhizosphere soil, root, stem, and leaf endosphere of field-grown poplar trees (*Populus tremula × Populus alba*) and applied 16S rRNA amplicon pyrosequencing to unravel the bacterial communities associated with the different plant habitats.

**Results:**

We found that the structural variability of rhizosphere microbiomes in field-grown poplar trees (*P. tremula × P. alba*) is much lower than that of the endosphere microbiomes. Furthermore, our data not only confirm microbiome niche differentiation reports at the rhizosphere soil–root interface but also clearly show additional fine-tuning and adaptation of the endosphere microbiome in the stem and leaf compartment. Each plant compartment represents an unique ecological niche for the bacterial communities*.* Finally, we identified the core bacterial microbiome associated with the different ecological niches of *Populus.*

**Conclusions:**

Understanding the complex host–microbe interactions of *Populus* could provide the basis for the exploitation of the eukaryote–prokaryote associations in phytoremediation applications, sustainable crop production (bio-energy efficiency), and/or the production of secondary metabolites.

**Electronic supplementary material:**

The online version of this article (doi:10.1186/s40168-017-0241-2) contains supplementary material, which is available to authorized users.

## Background

Inter-organismal associations between eukaryotic and prokaryotic organisms are one of the most studied research areas in (micro)biology in recent years. The massive interest in this topic is reflected by numerous studies ranging from the human microbiome [1, 2] and host–genotype associations therein [3] and gut microfauna of insects [4–6] to microbiota associated with plants [7–17]. In fact, most eukaryotes maintain close mutualistic relationships with microorganisms that are, in most cases, linked to their nutrient acquisition and thereby crucial for their performance and survival [18, 19]. Furthermore, the associated prokaryotic communities may play important roles in the regulation of the eukaryote immune system [20–23].

Plant–microbe interactions are of specific interest, not only to get a better understanding of their role during plant growth and development but also to allow exploitation of their relationships in phytoremediation applications, sustainable crop production, and the production of secondary metabolites [24–26]. The plant microbiome, often referred to as the host’s second or extended genome, comprises diverse microbial classes, including bacteria and archaea, fungi, oomycetes, and viruses. In its entirety, the plant microbiome represents one of the key determinants of plant health and productivity by providing a plethora of functional capacities [27–30]. More specifically, bacterial microbiota may improve nutrient bioavailability and transport from the soil as well as increase host tolerance to biotic (and abiotic stresses), promote stress resistance, and influence crop yield and quality. In return, the host plant delivers habitation and a constant supply of energy and carbon sources to the microbiota [29, 31]. Virtually all tissues of a plant host bacterial communities: at the soil–root interface (rhizosphere/rhizoplane), inside the plants tissues (root, stem, and leaf endosphere), and at the air–plant interface (phyllosphere environment). To a lesser extent, we can also distinguish the bacterial colonization of the anthosphere (flower) [32], the spermosphere (seeds) [33, 34], and the carposphere (fruit) [35]. All these microenvironments provide specific biotic and abiotic conditions for the residing bacterial communities.

Within plant–bacteria research, most attention has been dedicated to niche differentiation of bacterial communities at the rhizosphere soil–root interface [12, 14–16, 36–38]. For example, Gottel et al. compared the bacterial (and fungal) microbiota of mature poplar (*Populus deltoides*) trees using 16S ribosomal RNA (rRNA) gene pyrosequencing and revealed highly different root endophytic bacterial communities as compared to the rhizosphere soil [36]. Bulgarelli et al. [12] and Lundberg et al. [16] obtained qualitatively similar results in a study on the bacterial root microbiota of *Arabidopsis*. In contrast to the knowledge concerning the differentiation of the bacterial microbiome at the rhizosphere–endosphere barrier, a robust understanding of the structural composition of the bacterial microbiome present in different plant microenvironments and especially the relationship between below-ground and above-ground communities in field conditions has remained elusive. Recently, Coleman-Derr et al., Fonseca-Garcia et al., and Tardif et al. observed significant plant compartment effects respectively in the microbiome of *Agave* species, cacti, and willow [39–41]. Alternatively, Ottesen et al. reported significant differentiation of the epiphytic microbiome across different plant organs of tomato plants [42]. Other studies have focused on the leaf and root microbiomes [43, 44].

Here, we evaluate microbiome niche differentiation of bacterial communities associated with the rhizosphere soil and the root, stem, and leaf endosphere of field-grown wild-type poplar trees (*Populus tremula × Populus alba)* using 16S rRNA pyrosequencing. *Populus* is widely considered as the model of choice to study the biology of woody perennials and also provides an ideal model to explore the large variety of plant–microbe interactions [8, 9, 15, 36, 45–47]. Hybrid poplars are among the fastest growing trees and provide high economic flexibility with end-use applications such as the production of biofuels, pulp, and paper and other bio-based products such as chemicals and adhesives [48]. Furthermore, poplar trees can be grown on marginal land (land not suitable for food production) thereby evading the food versus fuel debate [49–51]. Sequencing of the poplar genome along with the availability of large natural provenances and breeding pedigrees, and the first successful use of gene editing have also opened biotechnological possibilities to accelerate breeding and genetic engineering [52–58]. In the present study, we focussed on two main questions: (i) How variable are bacterial communities associated with different field-grown trees within the same study site? (ii) Do bacterial communities in the endosphere differentiate among the plant niches, and how do they relate to the rhizosphere communities?

## Results

### Quality metrics of pyrosequencing analysis

Sequencing of the amplicon libraries resulted in a total of 341,915 raw reads prior to quality checking and assigning the reads to their respective sample. Average read length (± standard deviation) of reads before processing was 405 bp ± 96. After quality trimming and assigning reads to the different samples, 204,723 high-quality reads remained in the dataset with an average length (± standard deviation) of 207 bp ± 4 (Table 1).

**Table 1:** Quality metrics of pyrosequencing analysis

A. Total number of reads and read length before and after quality checking and trimming				
Total # of raw reads before QC	341,915			
Average read length before QC	405 ± 96			
Total # of assigned reads after QC	204,723			
Average read length after QC	207 ± 4			
B. Assigned reads	Rhizosphere soil	Root	Stem	Leaf
Average # of reads	5058 ± 615	5311 ± 643	2761 ± 1174	3034 ± 960
Singletons (%)	26.09 ± 0.01a	5.01 ± 0.55b	2.60 ± 0.35b	2.21 ± 0.65b
Normalization to 2000 reads per sample				
C. Non-target rRNA (%)	Rhizosphere soil	Root	Stem	Leaf
Chloroplast/plastid	0	0.01 ± 0.01	0.44 ± 0.17	0.03 ± 0.02
Mitochondria	0	0	0	0
Archaea	0.03 ± 0.01	0	0	0
D. Unclassified reads	Rhizosphere soil	Root	Stem	Leaf
Reads (%)	34.07 ± 1.10a	4.74 ± 0.32b	19.05 ± 4.32b	3.59 ± 1.03b

*A*: Quality metrics before and after quality control (QC), the average read length was calculated based on 52 samples across all plant compartments. *B*: Average number of assigned reads (± standard deviation) per plant compartment and percentages of singleton reads (± standard deviation). Numbers of singletons were statistically compared using one-way ANOVA and Tukey’s Honest significant differences post hoc tests. Statistical differences at the 95% confidence interval are indicated with lowercase letters. *C*: Comparison of the number of non-target 16S rRNA sequences (%) co-amplified during PCR amplification. and *D*: Reads that could not be unambiguously classified at the phylum level (“unclassified”) (%). Each plant compartment is evaluated separately and data represent 15 biologically independent replicates (± standard deviation) for the rhizosphere soil and root endosphere samples and 11 biologically independent replicates (± standard deviation) for the stem and leaf endosphere samples

Furthermore, we determined the co-amplification of non-target 16S rRNA (archaeal, chloroplast, and mitochondrial sequences) and the number of singletons identified within each plant compartment (%), as well as the number of reads that could not be unambiguously classified at the phylum level (Table 1). We found a distinct plant compartment effect in the retrieval of singletons. Significantly more singletons were obtained from the rhizosphere soil as compared to all other plant compartments (*F* (3, 44) = 454.7, *P* < 0.001) (Table 1). Under our optimized PCR conditions [9], no mitochondrial 16S rRNA sequences were co-amplified from any of the plant compartments. Minute fractions of chloroplast/plastidal 16S rRNA sequences were co-amplified from root, stem, and leaf samples (ranging from 0.01 to 0.44% of the normalized reads). Finally, in the rhizosphere, we identified a small portion of reads, which were assigned to the taxonomic domain *Archaea* (0.03%). In the rhizosphere soil, a large fraction of reads could not be unambiguously classified at the phylum level (34.07%). In the plant compartments, we were able to classify the majority of reads and only a relatively small proportion of reads remained unclassified (ranging from 3.59 to 19.05%). Unclassified reads at the phylum level were removed from the dataset for further analysis (Table 1).

### Alpha rarefaction curves and alpha diversity

**Fig. 1:**
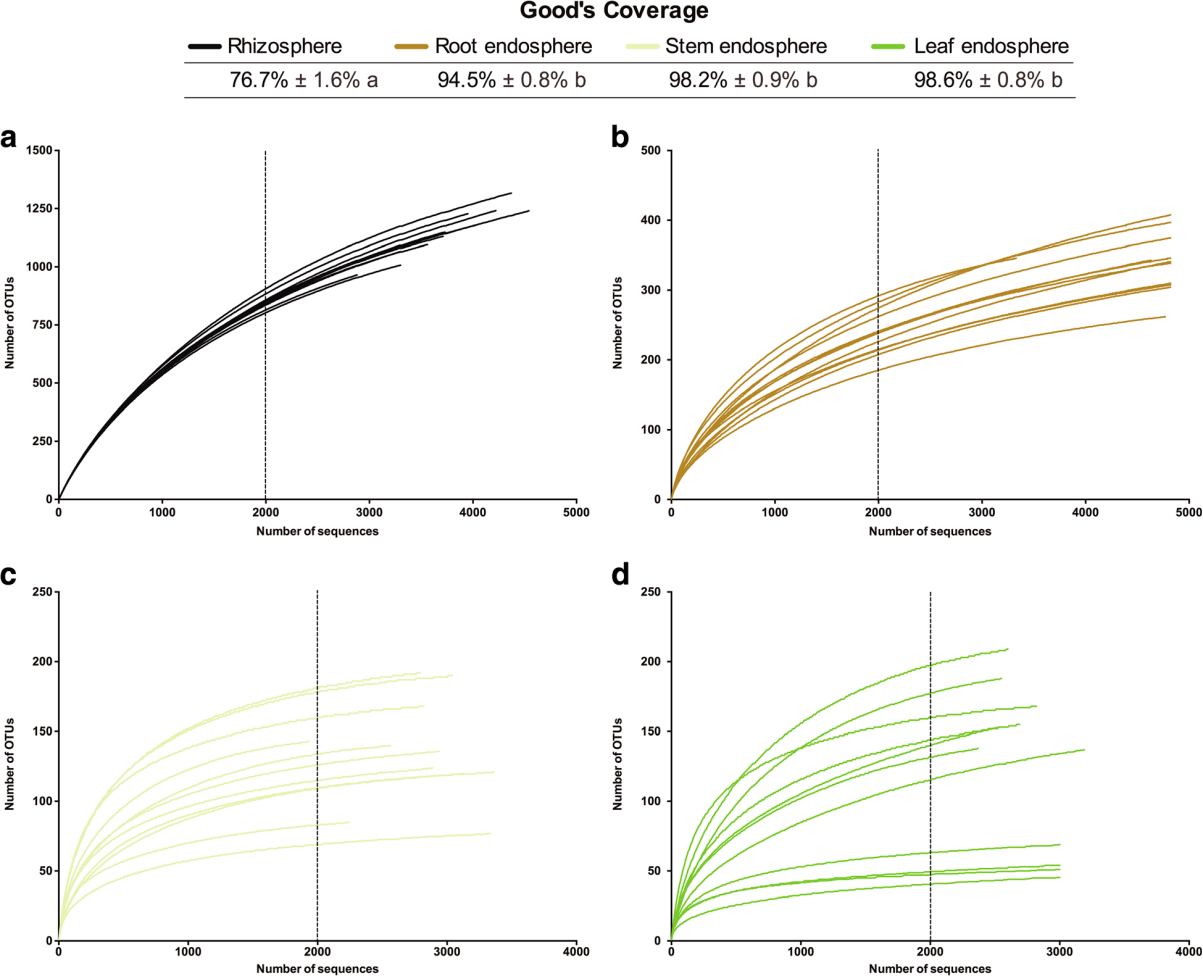
Average Good’s coverage estimates (%) and rarefaction curves of individual poplar trees per plant compartment (**a** rhizosphere soil, **b** root, **c** stem, **d** leaf). Good’s coverage estimates represent averages of 15 independent, clonally replicated poplar trees (rhizosphere soil and root samples) and 11 replicates (stem and leaf samples) (± standard deviation) and were calculated in mothur based on 10,000 iterations. *Lowercase letters* represent statistical differences at the 95% confidence interval (*P* < 0.05). Rarefaction curves were assembled showing the number of OTUs, defined at the 97% sequence similarity cut-off in mothur, relative to the number of total sequences. The *dashed vertical line* indicates the number of sequences subsampled from each sample to calculate alpha diversity estimates (Fig. 2)

**Fig. 2:**
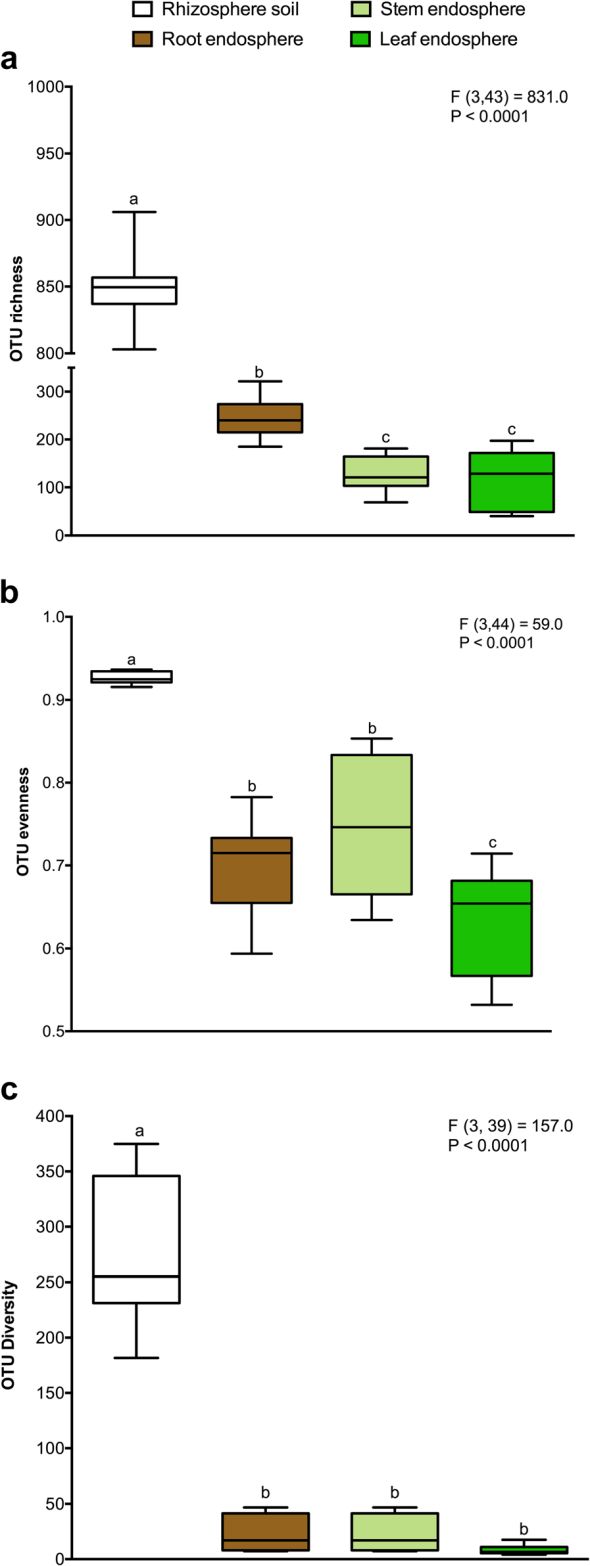
Alpha diversity estimates of the bacterial communities. **a** OTU richness estimates (number of observed OTUs). **b** Pielou’s evenness estimates. **c** Inverse Simpson diversity indices. Box plots display the first (25%) and third (75%) quartiles, the median and the maximum and minimum observed values within each data set. Alpha diversity estimates represent 15 biological replicates for the rhizosphere soil and root samples and 11 replicates for the stem and leaf samples and were calculated in mothur with 10,000 iterations. Data were analyzed by means of one-way ANOVAs and Tukey-Kramer post hoc comparisons. The overall plant compartment effects (F(DFn, DFd) and *P* value) are displayed at the top of each graph. Significant differences (*P* < 0.05) across plant compartments are indicated with *lowercase letters*

To construct alpha rarefaction curves (Fig. 1) and estimate differences in the alpha diversity (Fig. 2), we removed singletons (OTUs with only one sequence) from the dataset since these singletons could be due to sequencing artefacts. Rarefaction curves were constructed for each individual sample showing the number of observed OTUs, defined at a 97% sequence similarity cut-off in mothur [59], relative to the number of total identified bacterial rRNA sequences (Fig. 1). As expected, endophytic bacterial communities (Fig. 1b–d) were much less diverse than rhizospheric communities (Fig. 1a). Furthermore, the endophytic samples exhibited a higher degree of variation in the shape of their rarefaction curves as compared to the rhizospheric samples. Rarefaction curves evaluating the OTU richness per sample generally approached saturation. The majority of the root endophytic samples saturated around 250–300 OTUs and around 50–150 OTUs for the stem and leaf samples. The rhizospheric samples only showed saturation at about 1250 OTUs. Statistical differences in OTU richness were inferred from alpha diversity measures (Fig. 2). To further assess the sequencing depth, we calculated Good’s coverage scores in mothur based on 10,000 iterations (Fig. 1). Good’s coverage scores were highly comparable for all endosphere compartments (root, stem, leaf) ranging from 94.5 to 98.6% indicating that the sequencing depth was adequate to reliably describe the bacterial microbiome associated with these plant compartments. Good’s coverage scores of the rhizosphere soil data were significantly lower (*P* < 0.05) (76.7% ± 1.6%) as compared to those of the endosphere compartments. Rarefaction curves of the rhizosphere soil were starting to level off, but sequencing at a greater depth could have revealed more OTUs [see Additional file 1, Boneh and Efron estimator].

Alpha diversity, the microbial diversity within each sample, was analyzed based on the OTU richness, the inverse Simpson diversity index, and Pielou’s evenness (Fig. 2). To control for differences in sampling effort across plant compartments, we rarefied each sample to 2000 sequences per sample before calculating the diversity indices. OTU richness was highly dependent on plant compartment (*P* < 0.05) with high richness values for rhizosphere soil (848.9 ± 7.9) and consistently decreased richness estimates in the root samples (243.7 ± 9.6) and stem samples (126.7 ± 11.9). OTU richness indices of the leaf samples (118.3 ± 17.2) were comparable with those of the stem samples. For diversity and evenness estimates, we found a clear separation between the rhizosphere soil samples and endosphere samples (*P* < 0.05). Higher diversity and evenness measures were observed for the rhizosphere soil samples as compared to the samples of the endosphere plant compartments. In contrast, all endosphere compartments revealed highly comparable diversity and evenness estimates. Furthermore, to control for bias in the used community estimators, alternative estimators were calculated which resulted in highly similar conclusions (see Additional file 1).

### Beta diversity

**Fig. 3:**
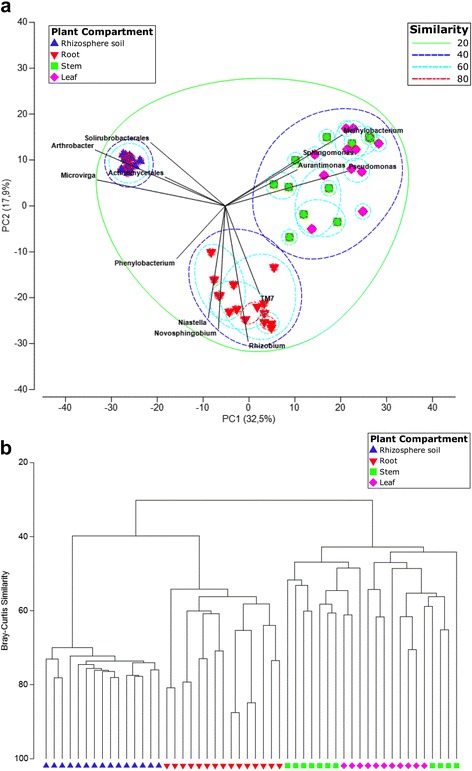
Plant compartment drives the composition of the bacterial communities at the OTU level. **a** Principle component analysis (PCA) of square-root transformed samples based on rarefaction to 2000 reads per sample. OTUs were defined at a 97% sequence similarity cut-off in mothur. OTUs differentiating the plant compartments are displayed as vectors on the PCA plots. **b** Hierarchical clustering (group average linkage) of the samples based on Bray–Curtis dissimilarity. Similarities based on Bray–Curtis (**b**) were superimposed on the PCA plot. PCA and hierarchical clusters were based on 15 biological replicates (rhizosphere soil and root samples) and 11 biological replicates (stem and leaf samples) and were constructed in PRIMER 7 with 10,000 iterations

We evaluated beta diversity at two phylogenetic levels, the phylum level and the OTU level (OTUs defined at a 97% similarity cut-off). To compare the composition of identified community members within different plant compartments and identify main factors driving community composition, a Bray–Curtis dissimilarity matrix was calculated on normalized (2000 sequences per sample) and square-root transformed read abundance data. Overall similarities in bacterial community structures among samples were displayed using principal component analysis (PCA). Furthermore, we also constructed a hierarchical clustering based on Bray–Curtis dissimilarities (Fig. 3).

PCA analyses revealed strong clustering of bacterial communities according to the different plant compartments (rhizosphere soil, root, stem, leaf) at each phylogenetic level (Fig. 3a and Additional file 2, left panel). At the OTU level, PC1 explained 32.5% and PC2 17.9% of the total variation (Fig. 3a). This pattern was recapitulated by hierarchical clustering of pairwise Bray–Curtis dissimilarities (Fig. 3b). Hierarchical clustering (at the OTU and phylum level) revealed complete clustering according to plant compartment for the rhizosphere soil and root samples (Fig. 3b and Additional file 2, right panel). The stem and leaf samples were clearly distinguished from rhizosphere soil and root samples but did not cluster completely according to their respective plant compartment. To statistically support the visual clustering of the bacterial communities in the above PCA analyses, different plant compartments were examined using ANOSIM (an analog of univariate ANOVA) with the Spearman rank correlation method (Table 2). All plant compartments rendered bacterial microbiota significantly dissimilar from each other (*P* values listed in Table 2) at the phylum and OTU level (see Additional file 3).

**Table 2:** Analysis of similarity (ANOSIM)

Phylogenetic level	Phylum		OTU	
ANOSIM output	*R*	*P*	*R*	*P*
Rhizosphere soil vs root	0.580	0.0001***	0.945	0.0001***
Rhizosphere soil vs stem	0.780	0.0001***	0.965	0.0001***
Rhizosphere soil vs leaf	0.819	0.0001***	0.992	0.0001***
Root vs stem	0.437	0.0001***	0.804	0.0001***
Root vs leaf	0.370	0.0003**	0.888	0.0001***
Stem vs leaf	0.232	0.01*	0.294	0.002**

Plant compartment effects on the bacterial community structures were calculated using ANOSIM (analysis of similarities) with the Spearman rank correlation method in Primer 7 (10,000 permutations). Plant compartments (rhizosphere soil, root, stem, leaf) were a priori defined groups at two phylogenetic levels: phylum level and OTU level. Significance levels: *P ≤ 0.01; **P ≤ 0.001; ***P ≤ 0.0001. *R*, ANOSIM test statistic. Graphical results of ANOSIM are displayed in Additional file 3

### Top members of the bacterial microbiome within each plant compartment

**Fig. 4:**
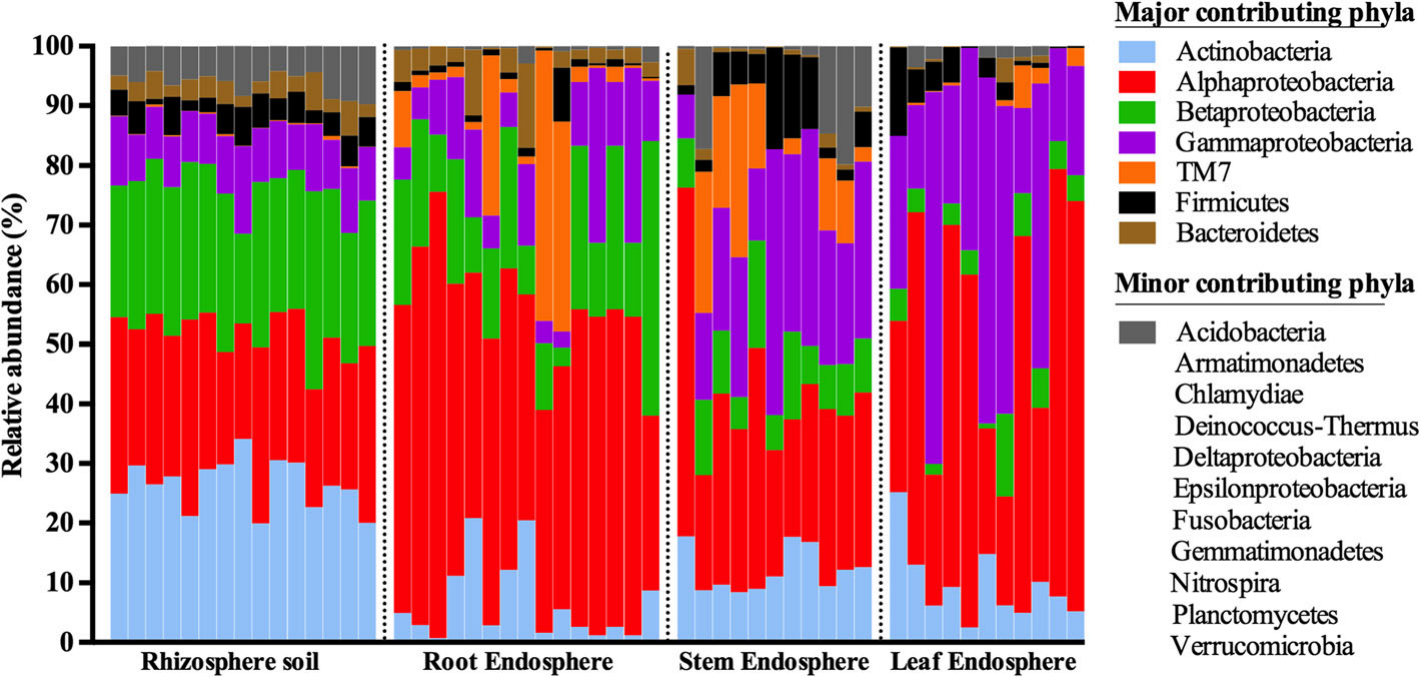
Phylum distribution of the OTUs. Relative sequence abundance of bacterial phyla associated with the rhizosphere soil and the root, stem and leaf endosphere. Proteobacteria OTU has been replaced by 5 OTUs at the subclass level (alpha, beta, delta, epsilon, gamma). Biological replicates (15 replicates for the rhizosphere soil and root samples and 11 replicates for the stem and leaf samples) are displayed in separate stacked bars. Major contributing phyla are displayed in different colours and minor contributing phyla are grouped and displayed in grey. Total relative abundances of all phyla and significant effects across plant compartments are listed in Additional file 4

Finally, we took a closer look at the individual bacterial phyla and OTUs, which differentiate the bacterial communities in the plant compartments. At the phylum level, we evaluated all observed phyla with ANOVA to test the effects of plant compartment (rhizosphere soil vs root vs stem vs leaf) on their relative abundance (%) (Fig. 4 and Additional file 4). The ANOVA model was [OTU] ~ compartment and included all four plant compartments followed by Tukey’s honest significant differences post hoc tests. Virtually all identified bacterial phyla displayed a significant plant compartment effect with the exception of *Armatimonadetes* (*P* = 0.27), *Chlamydiae* (*P* = 0.33), *Fusobacteria* (*P* = 0.11), and *Epsilonproteobacteria* (*P* = 0.33). In the rhizosphere samples, we observed a significant enrichment (*P* < 0.05) of *Actinobacteria* (relative abundance = 27.19%) and to a minor extent *Deltaproteobacteria* (1.90%), *Acidobacteria* (1.81%, not significantly different with the stem samples), *Nitrospira* (0.69%), *Gemmatimonadetes* (0.11%), and *Planctomycetes* (0.03%), as compared to the endosphere compartments. *Alphaproteobacteria* were significantly depleted in the rhizosphere soil samples (25.17%) as compared to the other plant compartments, although we still observed a high relative abundance in the rhizosphere soil compartment. *Betaproteobacteria* were significantly (*P* < 0.05) enriched in the rhizosphere soil (24.84%) and the root samples (15.56%) whereas *Gammaproteobacteria* were depleted in these compartments (rhizosphere soil = 9.62%; root = 7.23%) as compared to the stem and leaf samples. Candidate division TM7 was significantly enriched (*P* < 0.05) in the root (14.49%) and stem samples (10.29%) as compared to the rhizosphere soil and the leaf samples. Specifically for candidate division TM7, we observed very high variability in abundance from sample to sample in the root (ranging from 46% to as low as 0.46%) and stem endosphere (ranging from 29% to as low as 0%). Finally, in the stem samples, we observed a significant enrichment of *Deinoccus*–*Thermus* (3.37%) as compared to the other plant compartments. Total relative abundances of all phyla and significant effects across plant compartments are listed in Additional file 4.

**Fig. 5:**
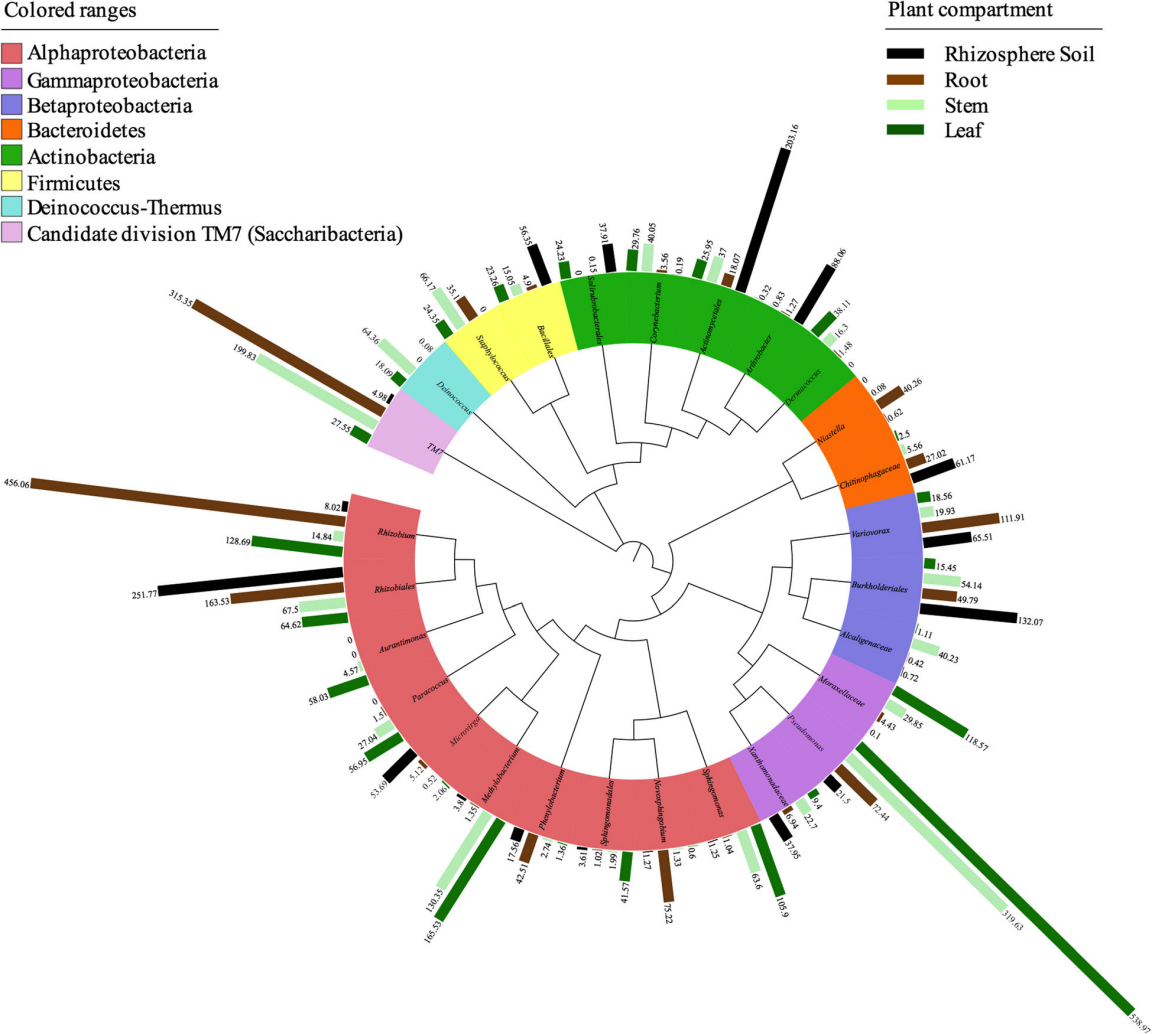
Top OTU members of the bacterial microbiome associated with the plant niches. Taxonomic dendrogram showing the core bacterial microbiome of each plant compartment. Color ranges identify phyla within the tree. *Colored bars* represent the relative abundance of each OTU in the plant compartments. Taxonomic dendrogram was generated with one representative sequence of each OTU using Unipro UGENE and displayed with the use of iTOL (Interactive Tree Of Life). Total relative abundances of all OTUs and significant effects across plant compartments are listed in Additional file 5

For the OTUs, we defined the core bacterial microbiome as the 10 most abundant OTUs of each of the plant compartments resulting in 27 OTUs altogether (Fig. 5 and Additional file 5). The percentages of the total community covered by the core OTUs ranged from 53% (rhizosphere soil), to 71% (root), to 63% (stem) and 77% (leaf). ANOVA was used to test the effect of plant compartment on the normalized sequence counts of members of the core community. The ANOVA model was [OTU] ~ compartment and included all four plant compartments followed by Tukey’s honest significant differences post hoc tests. We observed significant plant compartment effects across all identified core bacterial OTUs with the exception of *Solirubrobacterales* (*P* = 0.06) and *Phenylobacterium* (*P* = 0.38). In the rhizosphere soil, we observed a significant enrichment (*P* < 0.05) of *Actinomycetales* (10.16%), *Burkholderiales* (6.60%), *Arthrobacter* (4.40%), *Chitinophagaceae* (3.06%), *Bacillales* (2.82%), and *Microvirga* (2.68%) as compared to the endosphere compartments. In the root samples *Rhizobium* (22.80%), *Variovorax* (5.60%), *Novosphingobium* (3.76%), and *Niastella* (2.01%) were significantly enriched (*P* < 0.05) as compared to the other plant compartments. As described above, candidate division TM7 was significantly enriched in the root and stem samples as compared to the rhizosphere soil and leaf samples. *Rhizobiales* were significantly (*P* < 0.05) depleted in the stem (3.38%) and leaf samples (3.23%) whereas *Pseudomonas* (stem = 15.98%; leaf = 26.95%), *Methylobacterium* (stem = 6.52%; leaf = 8.28%), and *Sphingomonas* (stem = 3.19%; leaf = 5.29%) were enriched in these compartments as compared to the rhizosphere soil and root samples. Furthermore, in the stem samples, we found a significant (*P* < 0.05) enrichment of *Deinococcus* (3.21%), *Alcaligenaceae* (2.01%), and *Corynebacterium* (2.00%) as compared to the other plant compartments. Finally, in the leaf samples, we observed a significant (*P* < 0.05) enrichment of *Moraxellaceae* (5.93%), *Aurantimonas* (2.90%), and *Sphingomonadales* (2.08%). The total relative abundances of all core OTUs and significant effects across plant compartments are listed in Additional file 5.

To support the ANOVA results at OTU level and further ascertain which OTUs are responsible for the observed community differentiation between the plant compartments, we used species indicator analyses to discover significant associations between OTUs and plant compartments. Indicator analyses were performed on full community matrices and not only core OTUs to uncover effects possibly missed by the core OTU analysis. Full lists of indicator OTUs and their corresponding indicator values can be found in Additional file 6. Species indicator analysis revealed 94 indicator OTUs in rhizosphere soil, 18 in the root endosphere, 5 in the stem endosphere, and 9 in the leaf endosphere samples (see Additional file 6). However, when we used a community matrix excluding OTUs with an average relative abundance of >1%, we found 6 indicator OTUs in the rhizosphere soil (*Arthrobacter*, *Nitrospira*, *Norcardioides*, *Hyphomicrobiaceae*, *Mycobacterium* (*P* < 0.01) and *Microvirga* (*P* < 0.05)), 2 in the root samples (*Novosphingobium* and *Niastella*, *P* < 0.05), 2 in the stem samples (*Alcaligenaceae* and *Amnibacterium*, *P* < 0.05), and 2 in the leaf samples (*Sphingomonadales* and *Aurantimonas*, *P* < 0.05) (Table 3).

**Table 3:** Indicator species analysis

OTU (Genus or higher)	Plant compartment	Indicator value	*P*	Relative abundance (%)
*Arthrobacter*	Rhizosphere soil	0.978	0.0015**	4.403
*Nitrospira*	Rhizosphere soil	0.977	0.0024**	1.040
*Nocardioides*	Rhizosphere soil	0.970	0.0028**	1.117
*Hyphomicrobiaceae*	Rhizosphere soil	0.962	0.0036**	1.521
*Mycobacterium*	Rhizosphere soil	0.911	0.0068**	1.559
*Microvirga*	Rhizosphere soil	0.874	0.0119*	2.684
*Novosphingobium*	Root	0.981	0.0230*	3.761
*Niastella*	Root	0.960	0.0234*	2.013
*Alcaligenaceae*	Stem	0.886	0.0286*	2.205
*Amnibacterium*	Stem	0.830	0.0290*	1.104
*Sphingomonadales*	Leaf	0.937	0.0266*	2.079
*Aurantimonas*	Leaf	0.904	0.0270*	2.900

Associations were calculated with the Dufrene–Legendre indicator species analysis routine (Indval, indicator value) in R. Data table shows results for the analysis where rare OTUs (<1% relative abundance) were excluded. Significance levels: *P ≤ 0.05; **P ≤ 0.01. *P* values were corrected for multiple comparisons using the false discovery rate (FDR) with the Benjamini–Hochberg method. Full results of indicator species analysis are presented in Additional file 6

Finally, to provide a complete overview of the OTU distribution within the plant compartments, we calculated the proportion of OTUs uniquely identified in each specific plant compartment as well as the OTUs shared by the different plant compartments (Additional file 7). The proportion of OTUs shared by all plant compartments was 16.4%. Approximately 26% of all OTUs were exclusively found in the rhizosphere samples compared to the root samples (7.1%), the stem samples (3.2%), and the leaf samples (5.5%). Around 6% of the total OTUs were exclusively observed in the endosphere compartments. Finally, we clearly observed a higher overlap in OTUs from the rhizosphere soil samples and the root samples (11.7%) as compared to rhizosphere soil and the stem samples (1.8%) and the rhizosphere soil and leaf samples (2.8%).

## Discussion

### Quality of the pyrosequencing analysis

We used an optimized PCR approach to reduce co-amplification of chloroplast and mitochondrial 16S rRNA [9]. In many studies, the high homology between bacterial 16S rRNA genes, chloroplast 16S rRNA genes, and plant nuclear and mitochondrial 16S rRNA genes [60, 61] and moreover the high abundance of chloroplast 16S rRNA genes in these environments led to undesired co-amplification of non-target sequences [12, 15, 16, 36, 44]. Our optimized PCR approach resulted in very low co-amplification of these sequences and high retrieval of bacterial 16S rRNA sequences (Table 1). The highest retrieval of chloroplast 16S rRNA sequences was observed in the stem samples, corroborating results from our primer optimization [9] and reinforcing our view that the balance between the amount of endophytic bacterial DNA (bacterial cell density) and chloroplast DNA seems to play a more important role than the absolute chloroplast concentration. Finally, we also considered the number of singletons (sequences only found once in the dataset) obtained from each plant compartment (Table 1). Remarkably, we found high levels of singletons in rhizosphere soil and a decreasing number of singletons in other plant compartments. Singletons have been shown to comprise up to and beyond 60% of taxa in some surveys [62, 63] and are generally considered as being problematic since they represent inherently unreplicated data [64]. Most singletons arise from DNA sequencing errors (insertions, deletions, low-quality reads, inadequate clustering and formation of chimeric sequences) [65–68] creating false sequences and artificially inflating diversity estimates [69–71]. In our experimental setup, sequencing error (and potential creation of erroneous (singleton) sequences) is expected to be similar for all plant compartments with possibly a minor impact of carry-over contaminants in the rhizosphere soil samples, which could potentially increase PCR error. A confounding factor in this respect could be the use of different DNA extractions kits for the rhizosphere samples and the endosphere samples. Previously, we focused on extracting DNA from all the studied plant compartments (rhizosphere soil, root, stem, and leaf samples). [9], but we were unable to extract high-quality DNA (and quantity) from all four plant compartments using the same DNA extraction kit. To ensure high-quality and quantity DNA from all studied plant compartments and reduce bias from low DNA retrieval, we opted for a different DNA extraction kit for the rhizosphere soil samples and the endosphere samples. Nonetheless, a certain amount of bias may have been introduced in the results as a consequence of differences in the lysis efficiency of different DNA extraction kits [72, 73]. Notwithstanding these elements, our results indicate that the high discrepancy in the number of singletons between the plant compartments could in fact be attributable to more genuine rare (singleton) OTUs in the rhizosphere soil (Table 1). Indeed, the rhizosphere soil is renowned for its vast microbial diversity [74, 75]. For further analysis, we chose a conservative approach and treated all singletons as potentially erroneous and removed them from the data sets [68, 76]. However, the involvement of this rare biosphere in community dynamics and their ecological roles are largely unknown, but they could contribute to community stability by enabling fast responses to altering environmental conditions [77].

#### (i) How variable are bacterial communities associated with different field-grown trees?

We observed remarkably dissimilar shapes of the OTU rarefaction curves when comparing rhizosphere soil  and endosphere samples (Fig. 1). Rhizosphere soil samples displayed uniform rarefaction curves (Fig. 1a) whereas the variation in the shape of the rarefaction curves from the endophytic samples was much higher, especially for the stem and leaf samples (Fig. 1b–d). High variability of endophytic OTU richness, as depicted by the rarefaction curves, could possibly be caused by sporadic and non-uniform colonization of the roots and aerial plant compartments of *Populus* [36]. Gottel et al. attributed part of the variation to their inability to sequence the bacterial endophytic community deeply and uniformly enough because of the high co-amplification of organellar 16S rRNA (67,000 chloroplast and 65,000 mitochondrial sequences) [36]. However, our data exhibit roughly the same pattern without the co-amplification of non-target DNA (Table 1) and with high Good’s coverage estimates (Fig. 1). Therefore, our data suggest considerable variation in endophytic colonization as a major reason for the high variability in the rarefaction curves. Indeed, rhizosphere/rhizoplane colonization is primarily driven by (a) the deposition of large amounts of carbon (e.g., root exudates, mucilage by the root caps, etc.) by plants (rhizodeposition) and (b) the relatively simple or inelaborate chemo-attraction of the bacteria (and other microorganisms) to the root exudates [78–81]. Although, since root exudates and mucilage-derived nutrients attract a myriad of organisms to the rhizosphere environment, plant-associated bacteria have to be highly competitive to successfully colonize the root zone [82]. In contrast to rhizosphere/rhizoplane colonization, endophytic competence (i.e., ability to successfully colonize the host plant) can require specific traits (e.g., expression of genes involved in chemotaxis, the formation of flagella and pilli, the production of cell-wall degrading enzymes, etc.) and intricate interplay between rhizospheric soil-borne bacteria and the host plants innate immune system [12, 20, 25, 30, 82].

Furthermore, we also clearly observed more variation in the bacterial community structures in the endosphere as compared to the rhizosphere communities, especially in stem and leaf samples (Fig. 3 and Additional file 3). As mentioned previously, a possible confounding factor in the interpretation of these results is the use of different DNA extraction kits for the rhizosphere and endosphere samples. Nonetheless, the within group variation, as depicted by ANOSIM analysis (Additional file 3), of rhizosphere soil bacterial assemblages is very low. The soil biome is one of the richest microbial ecosystems on Earth with an estimated bacterial diversity of >2000 species within 0.5 g of soil [74, 75, 83]. Furthermore, the root exudation process is heterogeneous in space and time [84, 85]. Despite these factors, the formation of distinctive rhizosphere bacterial communities mediated by rhizodeposition (and chemo-attraction to photoassimilates) seems to be a very consistent and stable process across different poplar individuals. In contrast, variation within endophytic communities is much higher (Fig. 3 and Additional file 3). As mentioned previously, endophytic colonization and formation of stable communities appears to be a more variable process, as suggested by our results from the alpha rarefaction curves (Fig. 1), from the PCA analyses (Fig. 3a), the relative abundance of bacterial phyla (Fig. 4) and the ANOSIM results of the bacterial community structures (Additional file 3). Crucial factors underlining this variability are the nature of endophytic colonization and competence (e.g., bacterial motility, ability to produce cell-wall degrading enzymes) [25, 82], interplay with the host plants innate immune system [20]. and acute fluctuations in abiotic conditions (temperature, humidity, access to nutrients, etc.) which differ from the buffered fluctuations in the rhizosphere [31, 86]. However, in contrast, OTU richness and OTU diversity (Fig. 2) were markably higher in the rhizosphere soil as compared to the endosphere samples.

#### (ii) Do bacterial communities present in the endosphere differentiate within the plant niches, and how do they relate to the rhizosphere communities?

To control for differences in sampling effort across plant compartments, we rarefied each sample to 2000 sequences per sample, although rarefying and using linear models of abundance have been scrutinized recently by McMurdie and Holmes [87]. Initially, we estimated alpha diversity focussing on OTU richness, evenness and diversity. We found that richness estimates were highly dependent on plant compartment with rhizosphere soil, root and stem compartments clearly differentiated from each other by decreasing OTU richness (Fig. 2). These results are in concordance with the general views of endophytic colonization. Rhizodeposition and root exudation by the host plant in the root zone fuels chemo-attraction and colonization of the rhizosphere soil and rhizoplane, thereby leading to the formation of distinctive, highly rich, and diverse rhizosphere microbiomes [78–81]. After rhizoplane colonization, adaptation to an endophytic lifestyle is dependent on the ability of the soil-borne bacteria to pass (actively or passively) the endodermis and pericycle, reach the xylem vessels, and finally lead to systemic colonization of the plant [25, 82]. Systemic plant colonization by certain bacterial species is re-enforced by the proportion of OTUs shared by all the plant compartments (16.4%, Additional file 7). The rhizosphere soil–root interface acts as a selective barrier, and endophytic competence/colonization is limited to specific bacterial species. The great loss of diversity and evenness (Fig. 2a–c) from rhizosphere soil to endophytic compartments supports this view and indicates that only a limited number of bacteria can adapt to an endophytic lifestyle (loss of diversity) (Fig. 2c) and these bacterial strains will therefore dominate endophytic assemblages (loss of evenness) (Fig. 2b).

To compare the bacterial community structures present in the plant compartments, we clustered all samples using principal component analysis (PCA) and hierarchical clustering (Bray–Curtis dissimilarities) (Fig. 3). At the phylum level and OTU level, all samples strongly clustered according to plant compartment (*P* < 0.01) and rendered microbiota significantly dissimilar from each other (Fig. 3 and Table 2) (see Additional file 2). Again to put the results in a broader context, the caveat of using different DNA extraction kits for the rhizosphere samples and the endosphere samples may have introduced a certain amount of bias in these results. However, previously, we observed the same niche differentiation for the cultivable bacteria of poplar trees in the same field study [8]. Niche differentiation between rhizosphere and root endophyte microbiome has also been described for mature poplar trees growing in natural ecosystems (*P. deltoides*) [15, 36], for *Arabidopsis thaliana* [12, 16] and other plant species [10, 25, 37]. Recently, Bulgarelli et al. [31] proposed a two-step selection model for root microbiota differentiation from the rhizosphere where rhizodeposition and host genotype-dependent fine-tuning converge to select specific endophytic assemblages. Bulgarelli et al. argue that substrate-driven selection in the rhizosphere is expected to persist in the endosphere [31]. Indeed, our data suggest additional fine-tuning and niche differentiation of microbiota in the aerial plant organs (both at the phylum and OTU level), with the stem and leaf bacterial assemblages being remarkably dissimilar from the root and rhizosphere (Fig. 3 and Additional file 2) (Table 2). This in agreement with the studies of (a) Coleman-Derr et al. [39] and Fonseca-Garcia et al. [40], who revealed that the composition of bacterial communities in plants native to semi-arid and arid ecosystems (*Agave* species and cacti) were primarily determined by the plant compartment and (b) Tardif et al., who reported significant plant compartment effects in the willow microbiome [41]. Each of the plant microenvironments or ecological niches (rhizosphere soil, root, stem, and leaf) provide relevant biotic and abiotic gradients such as availability of soluble organic compounds [31, 88, 89]. This is further highlighted by the existence of specific proportions of OTUs, which were exclusively found in different plant compartments (e.g., 25.7% unique OTUs in the rhizosphere soil samples (Additional file 7)). The distribution of all identified OTUs across the different plant compartments (Additional file 7) also highlights several other aspects: (a) the inability of a large number of OTUs to colonize the plant (25.7% of all OTUs), (b) the existence of obligate endophytes which are only observed in the endosphere compartments (5.9% of all OTUs) and are strictly dependent on their host plant for survival [25], (c) the existence of facultative endophytes which may exist inside (endosphere) and outside the host plant (rhizosphere soil) [25], and (d) although most endophytic bacteria colonizing the host plant originate from the rhizosphere soil [82], some may originate elsewhere (e.g., colonization of the phyllosphere via aerosols and subsequently the leaf endosphere [90]) as evidenced by the proportion of OTUs uniquely identified in the leaf samples (5.5%).

### Drivers of microbiome niche differentiation

At the phylum level, *Actinobacteria* and *Proteobacteria* (mostly *Alpha*- and *Betaproteobacteria*) and to a lesser extent *Bacteroidetes*, *Firmicutes*, and *Acidobacteria* dominated the rhizobacterial assemblages. The ratio between *Proteobacteria* and *Acidobacteria* in rhizosphere bacterial communities has previously been shown to be an indicator of soil nutrient-content where *Proteobacteria* were linked to nutrient-rich soils and *Acidobacteria* to nutrient-poor soils [36, 91, 92]. Similarly to studies in *Arabidopsis* [12, 16], rice [14], and poplar [15, 36], the relative abundance of *Acidobacteria* and *Actinobacteria* decreased from the rhizosphere soil to the root microbiota whereas the relative abundance of *Proteobacteria* (mostly *Alpha*) increased in the root endosphere. Across different unrelated plant host species, the host-associated bacterial microbiota in the rhizosphere and root endosphere are consistently enriched with members belonging to the phylum *Proteobacteria* [12, 14–17, 36, 37, 93–95]. From our results, we can conclude that also the stem and leaf microbiota are dominated by *Proteobacteria* albeit with different OTU level members, mostly belonging to the *Alpha*- and *Gammaproteobacteria* (Figs. 4 and 5 and Additional files 4 and 5). The large overlap in key community members of endophytic bacterial assemblages across different plant host species demonstrates that endophytic competence (efficient colonization) and dealing with niche-specific plant settings (nutrient availability/variability, oxygen levels, etc.) is reserved for a minority of bacterial phyla. Enrichment and depletion of specific bacteria within the plant-associated microbiome are not passive processes but rather depend on active selection of microbial consortia by the plant host and/or opportunistic colonization of the available ecological niches by certain bacteria [14, 19, 31]. A remarkable phylum, candidate division TM7 (recently renamed phylum *Candidatus Saccharibacteria*), which has only been described from 16S rRNA gene sequence and genome data [96, 97], showed highly variable colonization capacities in the root and stem endosphere (Fig. 4 and Additional files 4 and 5). Phylum *Candidatus Saccharibacteria* is a highly ubiquitous phylum found in soils, sediments, wastewater, animals, and plant microbiomes [9, 12, 15, 97]. Furthermore, Shakya et al. also reported high variability in the relative abundance of phylum *Candidatus Saccharibacteria* (albeit in the rhizosphere microbiome of poplar) [15] possibly suggesting high sensitivity of these members to mild variations in abiotic and/or biotic stressors, strict nutritional requirements, variable responses of the plant’s innate immune system, strong influence of microbe-microbe interactions, or possible interactions with the plant host genotype.

Finally, at the OTU level (genus or higher), rhizosphere soil communities were dominated primarily by *Rhizobiales*, *Actinomycetales*, *Burkholderiales*, *Arthrobacter*, and *Variovorax* which were characteristically isolated from rhizosphere soil samples [10, 12, 36, 98]. Root endophytic assemblages were dominated by *Rhizobiales*, *Rhizobium*, and candidate division TM7 (with high variability). Dominant members of the stem samples are *Pseudomonas*, candidate division TM7, *Methylobacterium*, and *Deinococcus.* Finally, leaf samples mainly contained of *Pseudomonas*, *Sphingomonas*, and *Methylobacterium*. All of the above mentioned OTUs, which have been isolated from a variety of plant samples, may provide beneficial effects on plant health and growth [90, 99–102]. Remarkable in the stem (16%) and leaf endosphere (27%) is the efficient colonization capacity of *Pseudomonas* (Fig. 5 and Additional file 5). Niche-specific adaptation of *Pseudom​onas putida *has previously been described by Wu et al. [103]. We previously observed the same enrichment of *Pseudomonas* in the stem and leaf samples irrespective of the 16S rRNA primer pair used [9]. Since aerosol samples were found to harbor abundant *Pseudomonas* and *Sphingomonas* sequences [90], enrichment of these bacteria in the leaf endosphere may occur via dual origins, colonization of the rhizosphere and/or leaf stomatal colonization. Furthermore, *Sphingomonas* and *Methylobacterium*, both abundantly present in the leaf endosphere, were shown to harbor specific adaptation strategies such as TonB-dependent receptors to survive in the phyllosphere environment [100, 104, 105]*.*

## Conclusions

We proved that the structural variability of rhizosphere microbiomes in field-grown poplar trees (*P. tremula x P. alba*) is much lower than that of the endosphere microbiomes. The formation of rhizosphere bacterial communities appears to be a more stable and controlled process whereas endophytic colonization of the roots, stems, and leaves is highly variable. Furthermore, our data not only confirm microbiome niche differentiation reports at the rhizosphere soil–root interface but also clearly show additional fine-tuning and adaptation of the endosphere microbiome in the stem and leaf compartment. Each plant compartment represents an unique ecological niche for the bacterial communities*.* Future studies which include the analysis of specific host genotype effects (clones, genetically modified genotypes, etc.) could provide more insight into the plasticity or responsiveness of the bacterial communities to specific changes in the host plant. Finally, we identified a core bacterial microbiome associated with the different ecological niches of *Populus*. This could provide the basis for more detailed (isolation) studies of the identified abundant OTUs and gain further insight into the complex host–microbe interactions of *Populus.*

## Methods

### Field trial and sampling

A poplar field trial located in Ghent, Belgium, was selected to obtain samples for this study. This field trial was established in April 2009 and contains female poplar clones (*P. tremula* × *P. alba* cv. “717-1B4”). Poplars were micropropagated in vitro, and ramets were grown in soil in the greenhouse for 9 months. Thereafter, the stems were cut 10 cm above soil level, and plants were maintained for an additional 10 days in the greenhouse. Finally, coppiced trees were transferred to the field in May 2009. The trees were planted in a density of 15,000 trees per hectare with an inter-plant distance of 0.75 m [54, 106]. Poplar trees were sampled in October 2012. At the time of sampling, the height of the trees was on average approximately 3.5–4.5 m. Fifteen individual trees were sampled for the rhizosphere soil and root samples, and 11 trees were sampled for the stem and leaf samples. Collected samples included rhizosphere soil, roots, stems, and leaves. The root samples were collected at a depth of 5–10 cm below ground level. The rhizosphere soil was strictly defined as soil particles adhering to the roots. For the stem and leaf samples, one complete branch of each of the 11 poplar individuals was collected. Sampled branches were directly connected to the central trunk and had on average a circumference of approximately 4–7 cm and a height of approximately 80–140 cm To standardize and maximize reproducibility of the stem samples, several small stem “cores” with bark (5–7 cores; 1 cm each) were collected from each branch from the base to the top of the offshoot to represent the stem compartment. For the leaf samples, all leaves from the sampled offshoot were collected to represent the leaf compartment.

### Processing of samples

The samples were processed as described by Beckers et al. [8]. Briefly, the root samples were depleted from soil particles by shaking on a platform (20 min, 120 rpm). The soil particles directly dislodged from roots represented the “rhizosphere soil” compartment. Subsequently “root,” “stem,” and “leaf” compartments were cleared from epiphytic bacteria by sequential washing (surface sterilization) with (a) sterile Millipore water (30 s), (b) 70% (*v*/*v*) ethanol (2 min), (c) sodium hypochlorite solution (2.5% active Cl^−^ with 0.1% Tween 80) (5 min), and (d) 70% (*v*/*v*) ethanol (30 s) and finalized by rinsing the samples five times with sterile Millipore water. The plant samples were portioned into small fragments using a sterile scalpel and were subsequently macerated in a sterile phosphate saline buffer (PBS; 130 mM NaCl, 7 mM Na_2_HPO_4_, 3 mM NaH_2_PO_4_, pH 7.4) using a Polytron PR1200 mixer (Kinematica A6). Sterilization and homogenization of the plant samples were performed under aseptic conditions in a laminar airflow. Finally, quadruplicate aliquots of each sample (1.5 ml) of the homogenized plant material (root, stem, or leaf) were stored for all poplar individuals at −80 °C until DNA was extracted.

### DNA extraction

To minimize DNA extraction bias, DNA was extracted in quadruplicate from the rhizosphere soil, root, stem, and leaf samples [107, 108]. Approximately 250 mg of rhizosphere soil was used for each individual DNA extraction. DNA was extracted using the Power Soil DNA Isolation Kit following the protocol provided by the manufacturer (MoBio, Carlsbad, CA, USA). For the plant tissues, aliquots of homogenized plant material (1.5 ml) were first centrifuged (13,400 rpm, 30 min.) to collect all cells. Supernatants were discarded and DNA extractions were performed on pelleted plant material. DNA was extracted from plant samples using the Invisorb Spin Plant Mini Kit according to the manufacturer’s protocol (Stratec Biomedical AG, Birkenfeld, Germany).

### PCR amplification and 454 pyrosequencing

Quadruplicate DNA samples from all compartments were individually amplified using a Techne TC-5000 thermocycler (Bibby Scientific Limited, Staffordshire, UK). Based on previous optimization experiments with 16S rRNA primer pairs [9], we selected primer 799F (5′-AACMGGATTAGATACCCKG-3′), with three mismatches with the poplar chloroplast 16S rRNA, and primer 1391R (5′-GACGGGCGGTGWGTRCA-3′). Furthermore, we included negative controls to evaluate the presence of contaminating sequences in reagents, which were checked using gel-electrophoresis (1.5% agarose gel, 90 V, 30 min.). A first round of PCR amplification was conducted using these primers without the Roche 454 pyrosequencing adaptors and sample-specific barcodes. Each 25 μl PCR reaction contained approximately 10 ng of DNA and was carried out using the FastStart High Fidelity PCR System (Roche Applied Science, Mannheim, Germany). Each reaction contained 2.75 μl FastStart 10× reaction buffer, 1.8 mM MgCl_2_, 0.2 mM dNTP mix, 0.4 μM of each primer, and 2 U FastStart HiFi polymerase. Cycling conditions included initial denaturation at 94 °C for 3 min, followed by 35 cycles of denaturation at 94 °C for 1 min, annealing at 53 °C for 1 min, and extension at 72 °C during 1 min; a final extension phase was performed at 72 °C during 10 min. PCR amplicon pools were cleared from residual primers and primer dimers by separating the PCR products on a 1.5% agarose gel (90 V, 30 min.), excising the bacterial product (amplicon length = 592 bp) and extracting the DNA from the gel slices using the QIAQuick gel extraction kit (Qiagen Benelux N.V., Venlo, The Netherlands). Mitochondrial by-products (1000 bp) were eliminated via this gel-purification. Following the first round of PCR amplification and gel-purification of the PCR products, a second round of PCR amplification was performed with primer 967F (5′CAACGCGAAGAACCTTACC-3′)-1391R(5′-GACGGGCGGTGWGTRCA-3′) to reduce the amplicon length (424 bp) to a more suitable length for 454 pyrosequencing. The forward primer (967F) was fused to the Roche 454 pyrosequencing adaptor A including a sample-specific 10-bp barcode (multiplex identifiers, MIDs). The reverse primer (1391R) was fused to adaptor B (Roche Applied Science, Mannheim, Germany). PCR cycling conditions were identical as described above, except for the number of PCR cycles that was lowered to 25.

Subsequently, quadruplicate PCR amplicon pools from the corresponding samples were grouped together resulting in 15 samples (rhizosphere soil and root) and 11 samples (stem and leaf) per plant compartment (15 biological replicates × 2 plant compartments + 11 biological replicates × 2 plant compartments = total of 52 samples). PCR amplicon pools were purified to remove PCR primers and primer dimers using the QIAquick PCR purification kit (Qiagen Benelux B.V., Venlo, the Netherlands). Following purification, the quality of the amplicon pools was evaluated using an Agilent 2100 Bioanalyzer system (Agilent Technologies, Diegem, Belgium) according to the manufacturer’s protocol. Finally, purified amplicon libraries were quantified with the Quant-iT PicoGreen dsDNA Assay Kit (Invitrogen, Carlsbad, CA, USA) and a Fluostar Omega plate reader (BMG Labtech, Ortenberg, Germany) and pooled in equimolar concentrations. Rhizosphere samples (15) and root samples (15) were each separately pooled into two amplicon libraries. Stem and leaf samples were grouped into an additional library consisting of 22 samples (11 stem and 11 leaf samples). Each amplicon library (total of 3) was sequenced on one eighth of a Picotiter Plate on a Roche Genome Sequencer FLX+ using Titanium chemistry (Roche Applied Science, Mannheim, Germany) by Macrogen (Seoul, Korea).

### Sequence processing

Sequencing generated three individual standard flowgram format (SFF) files, which were analyzed separately using the software package mothur (version 1.33.2) following the standard operating protocol outlined in https://www.mothur.org/wiki/454_SOP [59]. Briefly, the sequencing error was reduced by denoising (shhh.flows, Mothur implementation of Amplicon Noise algorithm) and quality trimming, which removed reads shorter than 200 bases, reads with homopolymers longer than 8 bases, and reads containing ambiguous bases. Unique sequences were identified, while archiving the abundance data of the unique sequences, and aligned using align.seqs with the SILVA reference alignment (Release 119) [109]. Within the unique reads, chimeric sequences were identified using the Uchime tool [110] followed by their removal from the dataset. Unique sequences were classified using the mothur implementation of the Bayesian classifier where an 80% bootstrap cut-off value was used for assigning taxonomic classifications. Abundance data of sequences matching “Chloroplast” and “Mitochondria” were archived and these sequences were removed from the data sets. Subsequently, pairwise distances were calculated between all remaining unique sequences and a distance matrix was created. Average neighbor clustering was performed and, using a 0.03 OTU definition (97% sequence similarity cut-off level), a majority consensus taxonomy was obtained for each OTU. To minimize the impact of sequencing artefacts, we removed singletons from the datasets [64]. Subsequently, rarefaction curves were assembled, and Good’s coverage scores were calculated in mothur based on 10,000 iterations. To calculate diversity indices (richness, diversity, evenness) while controlling for the sampling effort, each sample was rarefied to 2000 sequences. OTU richness, corresponding to the number of observed OTUs per sample (sobs), inverse Simpson diversity indices [111], and Pielou’s evenness indices [112] were calculated in mothur based on 10,000 iterations. To exclude bias in the community richness, evenness, and diversity estimators, we included several alternative estimators (Additional file 1).

### Statistical analysis

Statistical analyses were performed in R 2.15.1 (The R Foundation for Statistical Computing, Vienna, Austria) [113]. Normal distributions of the data were checked with the Shapiro–Wilk test and homoscedasticity of variances was analyzed using either Bartlett’s or the Fligner–Killeen test. Significant differences in the variance of parameters were evaluated, depending on the distribution of the estimated parameters, either with ANOVA or the Kruskal–Wallis rank sum test. Post hoc comparisons were conducted by either the Tukey’s honest significant differences tests or pairwise Wilcoxon rank sum tests. ANOVA was used to test the effect of the plant compartment (rhizosphere soil, root, stem, leaf) on the read abundances. Hierarchical clustering (based on Bray–Curtis dissimilarities) and principal component analyses (PCA) were performed in and displayed with PRIMER 7 [114]. To statistically support the visual clustering of the bacterial communities in the PCA analyses, the different plant compartments were compared using permutation-based hypothesis tests: tests of the multivariate null hypotheses of no differences among a priori defined groups were examined using ANOSIM (an analog of univariate ANOVA) with the Spearman rank correlation method in PRIMER 7. Indicator species analysis was performed using the multipat function of the indicspecies package in R (version 1.7.1) [115]. *P* values were corrected for multiple comparisons using the false discovery rate (FDR) with the Benjamini–Hochberg method. Taxonomic dendrogram (Fig. 5) was generated with one representative sequence of each OTU using Unipro UGENE and displayed with the use of iTOL (Interactive Tree Of Life) [116].

## Additional files

Additional file 1: Community estimators. Values represent averages (±standard deviation) of 15 biological replicates (rhizosphere soil and root samples) and 11 replicates (stem and leaf samples) after normalization to 2000 sequences. Normal distributions of the data were checked with the Shapiro–Wilk test and homoscedasticity of variances was analyzed using either Bartlett’s or the Fligner-Killeens test. Significant differences in the variance of parameters were evaluated with ANOVA, and post hoc comparisons were conducted by the Tukey’s honest significant differences tests. Plant compartment effects show the overall ANOVA results: *F*(DFn, DFd) and *P* value. Significant differences at the 95% confidence interval (*P* < 0.05) between the plant compartments are indicated in lowercase letters (P).(XLSX 11 kb)
Additional file 2: Plant compartment drives the composition of the bacterial communities at phylum level. Left panel: principle component analysis (PCA) of square-root transformed samples based on rarefaction to 2000 reads per sample. OTUs were defined at a 97% sequence similarity cut-off in mothur. OTUs differentiating the plant compartments are displayed as vectors on the PCA plots. Right panel: hierarchical clustering (group average linkage) of the samples based on Bray–Curtis dissimilarity. Dissimilarities based on Bray–Curtis were superimposed on the PCA plot (left panel). PCA and hierarchical clusters were based on 15 biological replicates (rhizosphere soil and root samples) and 11 replicates (stem and leaf samples) and were constructed in PRIMER 7 with 10,000 iterations.(TIFF 5 kb)
Additional file 3: Graphical representation of the ANOSIM (analysis of similarities) analyses at the phylum (A) and OTU level (B) within each plant compartment (rhizosphere soil, root, stem, leaf). Box plots show the variation observed in the taxonomical composition of the different replicate samples of each plant compartment (leaf, rhizosphere soil, root, stem). Variation is based on the dissimilarity (using Bray–Curtis dissimilarities) of the samples (taxonomically) within each plant compartment as well as the overall dissimilarity between the different samples in the different plant compartments (noted as “Between”). Box plots display the first (25%) and third (75%) quartiles, the median (bold line), maximum and minimum observed values (without outliers). Outliers (more or less than 3/2 of the upper/lower quartile) are displayed as open circles. ANOSIM and resulting box plots were calculated based on 15 biological replicates (rhizosphere soil and root samples) and 11 replicates (stem and leaf samples) in R with 10,000 iterations. *R*-statistic and *P* values are displayed on top of each individual graph.(TIFF 3 kb)
Additional file 4: Plant compartment effect on the individual bacterial phyla. Values represent average number of reads (±standard error) and relative read abundances (%) based on 15 biological replicates (rhizosphere soil and root samples) and 11 replicates (stem and leaf samples) after normalization to 2000 sequences. Normal distributions of the data were checked with the Shapiro–Wilk test and homoscedasticity of variances was analyzed using either Bartlett’s or the Fligner–Killeens test. The ANOVA model was [OTU] ~ compartment and included all four plant compartments followed by Tukey’s honest significant differences post hoc tests. Plant compartment effects show the overall ANOVA results: *F*(DFn, DFd) and *P* value. Significant differences at the 95% confidence interval (*P* < 0.05) between the plant compartments are indicated in lowercase letters. *P* values were corrected for multiple comparisons using the false discovery rate (FDR) with the Benjamini–Hochberg method.(XLSX 40 kb)
Additional file 5: Top members of the bacterial microbiome of each plant compartment. Values represent average number of reads (±standard error) and relative read abundances (%) based on 15 biological replicates (rhizosphere soil and root samples) and 11 replicates (stem and leaf samples) after normalization to 2000 sequences. Normal distributions of the data were checked with the Shapiro–Wilk test, and homoscedasticity of variances was analyzed using either Bartlett’s or the Fligner–Killeens test. The ANOVA model was [OTU] ~ compartment and included all four plant compartments followed by Tukey’s honest significant differences post hoc tests. Plant compartment effects show the overall ANOVA results: *F*(DFn, DFd) and *P* value. Significant differences at the 95% confidence interval (*P* < 0.05) between the plant compartments are indicated in lowercase letters. *P* values were corrected for multiple comparisons using the false discovery rate (FDR) with the Benjamini–Hochberg method. The total amount of reads covered by the top members of the microbiome (top ten OTUS) in each plant compartment are indicated at the bottom. Gray colored values represent the top ten OTUs per plant compartment.(XLSX 42 kb)
Additional file 6: Indicator species analysis. Associations were calculated with the Dufrene–Legendre indicator species analysis routine (Indval, indicator value) in *R*. Significance levels: *P* ≤ 0.05•; *P* ≤ 0.01*; *P* ≤ 0.001**. *P* values were corrected for multiple comparisons using the false discovery rate (FDR) with the Benjamini–Hochberg method.(XLSX 44 kb)
Additional file 7: OTU distribution across the plant compartments. Venn diagram showing the overlap in operational taxonomic unit (OTU) composition between the different plant compartments.(TIFF 471 kb)
