# Supplementary figures and images for: Structural variability and niche differentiation in the rhizosphere and endosphere bacterial microbiome of field-grown poplar trees
Source: Microbiome. 2017 Feb 23;5:25. doi: 10.1186/s40168-017-0241-2 (PMC5324219; doi:10.1186/s40168-017-0241-2)

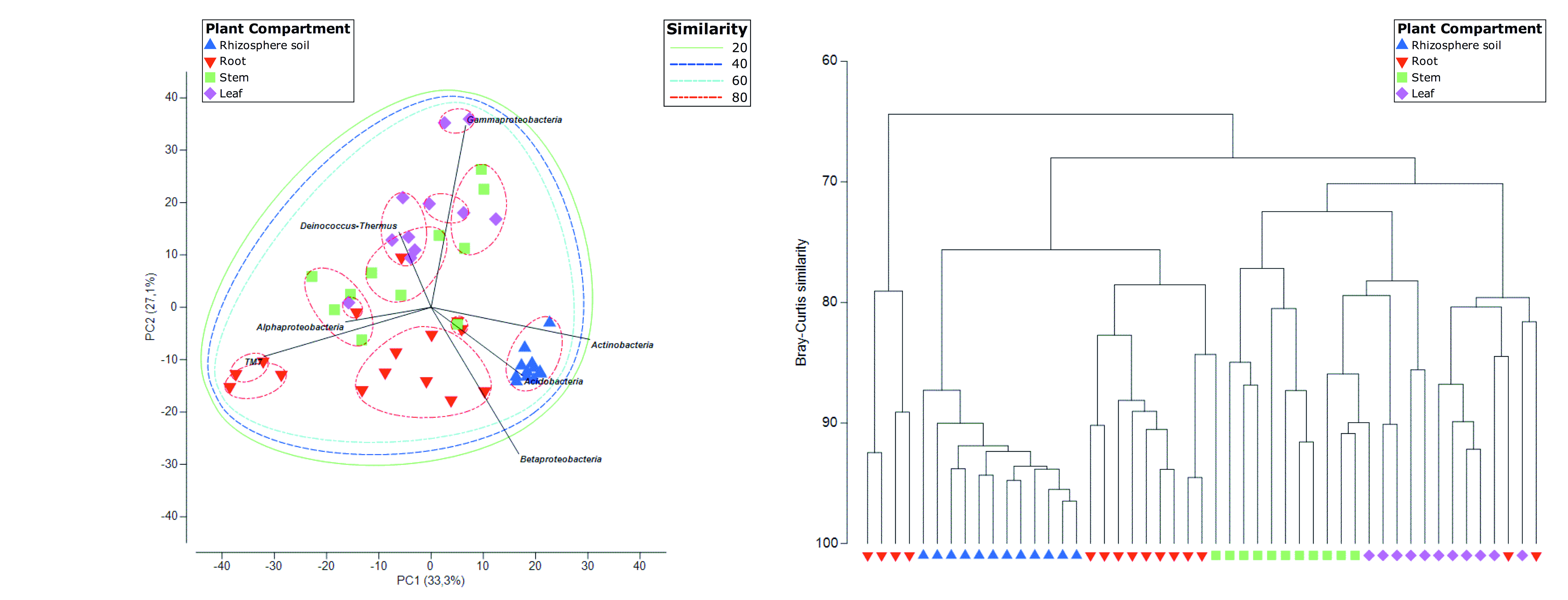

Supplement: Additional file 2: — Plant compartment drives the composition of the bacterial communities at phylum level. Left panel: principle component analysis (PCA) of square-root transformed samples based on rarefaction to 2000 reads per sample. OTUs were defined at a 97% sequence similarity cut-off in mothur. OTUs differentiating the plant compartments are displayed as vectors on the PCA plots. Right panel: hierarchical clustering (group average linkage) of the samples based on Bray–Curtis dissimilarity. Dissimilarities based on Bray–Curtis were superimposed on the PCA plot (left panel). PCA and hierarchical clusters were based on 15 biological replicates (rhizosphere soil and root samples) and 11 replicates (stem and leaf samples) and were constructed in PRIMER 7 with 10,000 iterations.(TIFF 5 kb) [file 40168_2017_241_MOESM2_ESM.tiff]

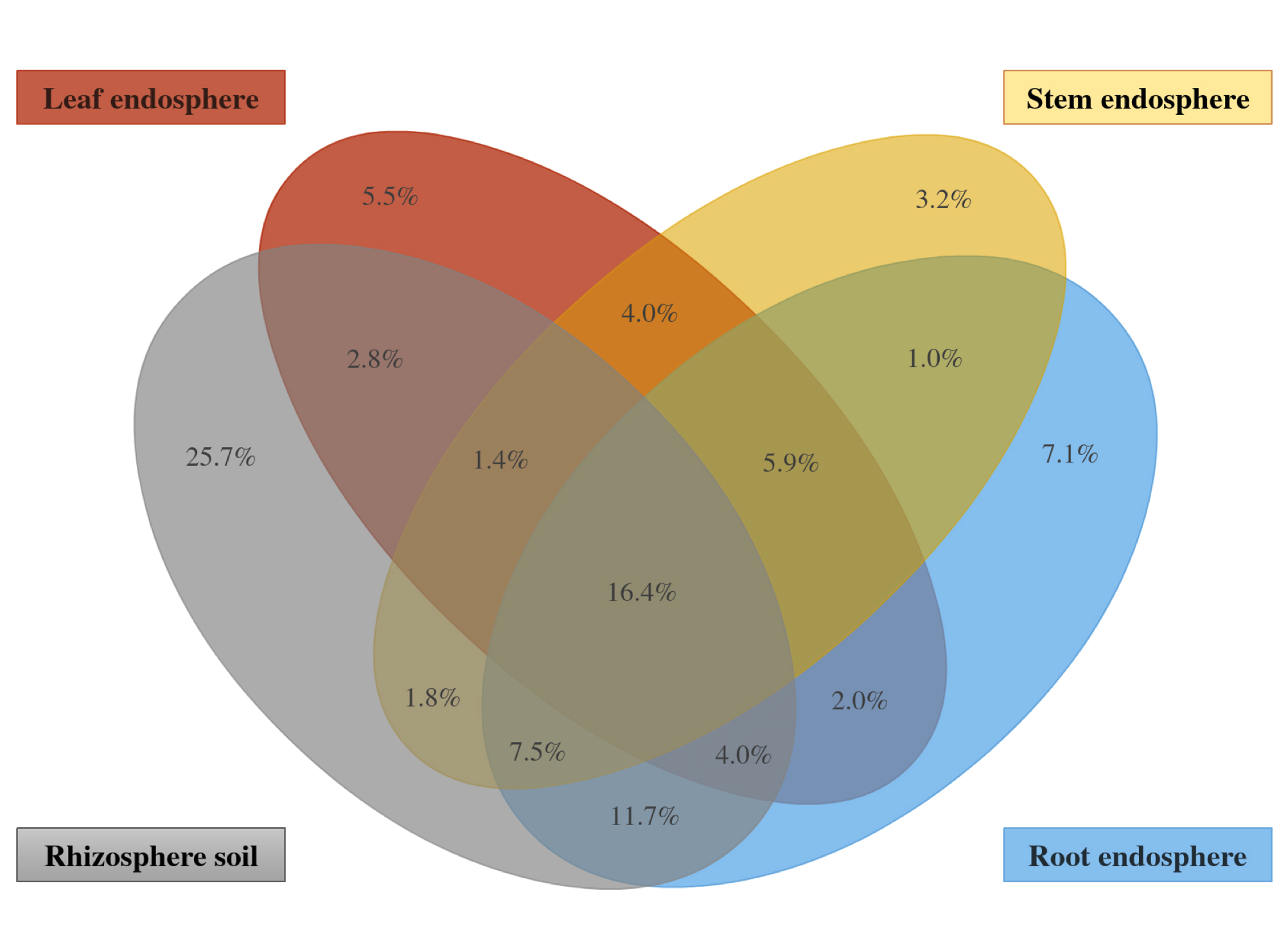

Supplement: Additional file 7: — OTU distribution across the plant compartments. Venn diagram showing the overlap in operational taxonomic unit (OTU) composition between the different plant compartments.(TIFF 471 kb) [file 40168_2017_241_MOESM7_ESM.tiff]
